# Measurement of Heat Transfer from Anodic Oxide Film on Aluminum in High Knudsen Number Flows

**DOI:** 10.3390/mi11030234

**Published:** 2020-02-25

**Authors:** Hiroki Yamaguchi, Kenji Kito

**Affiliations:** Department of Micro-Nano Mechanical Science and Engineering, Nagoya University, Nagoya 464-8603, Japan

**Keywords:** gas–surface interaction, thermal accommodation coefficient, vacuum

## Abstract

The heat transfer in vacuum depends on the gas–surface interaction. In this study, the heat flux from anodic oxide films on aluminum with different anodizing times through a gas confined between two surfaces with different temperatures was studied. We prepared a non-treated surface, a surface with a normal anodizing time of 30 min, and a surface with 90 min, where the formed film would partially dissolve by long time exposure to the solution. The formation of the films was checked by electrical resistance. Scanning electron microscope (SEM) images were obtained for the three sample surfaces. Even though it was difficult to observe the hexagonal cylindrical cell structures on anodic oxide films, the 30 min sample surface was shown to be rough, and it was relatively smooth and powdery for the 90 min sample surface. The heat fluxes from three sample surfaces were measured from the free-molecular to near free-molecular flow regimes, and analyzed to obtain the energy accommodation coefficients. The heat fluxes were well fitted by the fitting curves. The energy accommodation coefficients for both helium and argon increased by anodizing an aluminum sample surface, while they decreased with increasing the anodizing time up to 90 min indicating the dissolution of the film.

## 1. Introduction

The heat transfer from a hot to a cold surface in vacuum is a basic problem. In vacuum, the Knudsen number is large due to a large mean free path of gas molecules. In such a high Knudsen number flow, the number of collisions between gas molecules and a solid surface cannot be neglected compared with that between gas molecules. Then, the gas–surface interaction plays an important role in the heat transfer problem.

The gas–surface interaction, which is a scattering process of gas molecules from a solid surface at the boundary of a thermal-fluid field, is known to be a complicated process depending on many parameters of gas species and a solid surface [1]. As the boundary condition of a thermal-fluid field, the statistical behavior of molecules is important and convenient for analysis. For such purpose in the gas–surface interaction, the accommodation coefficient [2], which is an integral characteristics of the interaction, is often employed in models for the gas–surface interaction. The accommodation coefficient represents mean transfer rate, probability, fraction or efficiency of exchanging physical properties between gas molecules and a solid surface through the interaction. For the heat transfer problem, energy transfer is related; thus, we focus on the energy accommodation coefficient (EAC) or the thermal accommodation coefficient, which are equivalent for a static equilibrium gas. The EAC α is defined as [1,2,3]

(1) $$\alpha =\frac{E_{i}-E_{r}}{E_{i}-E_{s}},$$

where *E_i_*, *E_r_* and *E_s_* are the mean incident and reflected energy fluxes, and the energy flux of gas molecules fully accommodated to the surface, respectively.

The EACs or the thermal accommodation coefficients for various pairs of gas species and surface materials have been measured for a long time [2]. Although there are quite large scatterings in the measured values for gas–surface pairs, several qualitative characteristics are discussed. For the effect of the surface roughness, it is known to have a large EAC for a rough surface, i.e., gas molecules accommodate well to a rough surface, because of multiple collisions [1]. Therefore, engineering surfaces have been considered to have the EAC around unity. Recent study [4] showed the effect of the surface roughness on the EAC by comparing the results on the machined, polished and deposited surfaces with or without plasma treatment, showing that the surface roughness appeared to have only a minor effect. The rms surface roughness of these surfaces was reported as ~2 μm for the machined surfaces and ~0.02 μm for the polished surfaces.

In this study, the effect of surface roughness on the energy accommodation coefficient is studied by employing an anodic oxide film on aluminum. An anodic oxide film has the hexagonal cylindrical cell structure with several to several hundreds nm diameter [5,6]. The structure with many pores of such size, similar to porous materials, may cause multiple collisions of gas molecules. Therefore, it is suited to see the effect of roughness on the EAC. It is also important to note that the anodization process is a wet process; thus, the process would roughen all the area of sample surfaces even though there are distortions, large adsorbates or dimples on sample surfaces.

## 2. Materials and Methods

The heat flux from a sample surface of an anodic oxide film on aluminum to a cold vacuum chamber in vacuum as a function of pressure was measured to extract the EAC.

### 2.1. Sample Surfaces

The sample surfaces of the anodic oxide film were prepared by anodizing a 0.3-mm-thick aluminum plate (A1050P, AS-ONE, Osaka, Japan), which is a general purpose product. A strip was cut from the plate. Then, it was wiped by acetone, dipped in a solution of NaOH, and rinsed in distilled water. The strip was then immersed in a diluted solution of H_2_SO_4_ (1 mol/L), and the electric current was applied with the formation voltage of 20 V and the current density of 12.5 mA/cm^2^. Chilled water was circulated through a coil placed in the solution to keep its temperature constant at around 5 °C. The anodization time is usually set to 10–30 min for the above conditions [5,6]. It is known that with a long anodization time a hexagonal cylindrical cell structure of an anodized film is chemically dissolved by long time exposure to the solution. To see the effect of the cell structure, we selected 30 min for a normal sample and 90 min for less roughened surface with the anodized material. These two sample surfaces were compared with a non-treated sample surface, which is hereafter called the 0 min sample surface.

First, electrical resistance of the sample surfaces was measured by a tester to check the formation of anodized films on the sample surfaces, since an anodic oxide film is an insulator. It was easily verified that anodized films were formed on both the 30 min and 90 min samples.

**Figure 1:**
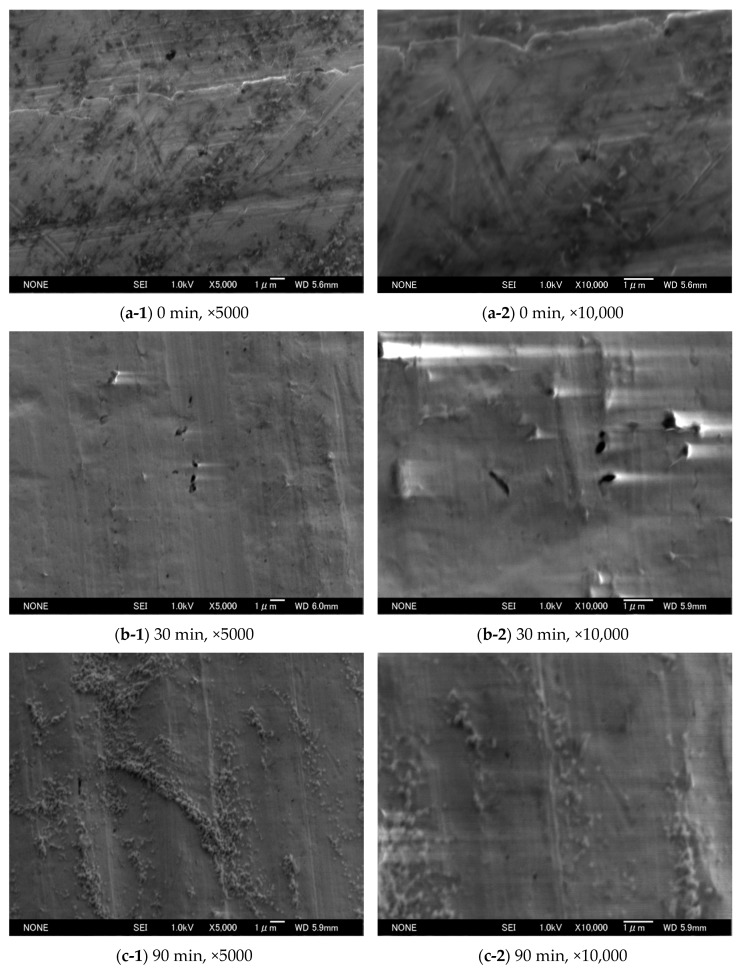
Scanning electron microscope (SEM) images of the anodic oxide films on the sample surfaces: (**a**) 0 min; (**b**) 30 min; and (**c**) 90 min with the magnification of (**1**) ×5000; and (**2**) ×10,000.

To check the surface roughness in detail, the sample surfaces were measured by SEM (JSM-7000F, JEOL, Tokyo, Japan). The obtained scanning electron microscope (SEM) images are shown in Figure 1. We tried to obtain high magnification images; however, it was difficult to observe nano-scale cell structures of the anodic oxide film because of the nature of the film. From these images, it is easily observed that the sample surfaces are quite rough. There are many scratches and roughness already in the 0 min sample surface. Several quite large bumps or dents (black area) appeared on the 30 min sample surface, while small scratches are relatively smoothened. On the other hand, the 90 min sample surface becomes relatively smoother than the 30 min sample surface, but powdery. This might be the result of the dissolution of the film from the top by long time exposure to the solution [5,6] as mentioned above. It is also interesting to see that there are fewer bumps or dents that are observed in the 30 min sample surface.

### 2.2. Method

In the free-molecular flow regime, the heat flux between two surfaces with different temperatures is explained by the energy transfer by molecular motions. The heat flux in the free-molecular flow regime $q_{FM}$ is theoretically expressed as

(2) $$q_{FM}=\frac{1}{8}\alpha \frac{\gamma +1}{\gamma -1}\frac{\bar{v}}{T}p\Delta T,\;\bar{v}=\sqrt{\frac{8kT}{\pi m}},$$

where γ, $\bar{v}$, T, p, ΔT, k and m are the specific heat ratio, the mean molecular speed, temperature, pressure, the temperature difference of two surfaces, the Boltzmann constant, and the molecular mass of the gas, respectively. The heat flux is proportional to pressure and the EAC; thus, by measuring the heat flux as a function of pressure, the EAC can be derived.

However, as mentioned in our previous studies [7,8,9], it was not easy to accurately measure only in the free-molecular flow regime, i.e., at a high vacuum condition, in a simplified low-cost apparatus because of small heat flux. Therefore, we used pressure conditions slightly higher than the upper limit of the free-molecular flow regime. A general model expression to describe the heat flux from the free-molecular flow regime up to the continuum flow regime was employed [10,11], which is expressed as

(3) $$\frac{1}{q}=\frac{1}{q_{FM}}+\frac{1}{q_{C}},$$

where $q_{C}$ is the heat flux in the continuum flow regime. The heat flux as a function of pressure would be slightly curved by this expression. The obtained heat flux was fitted by this expression to obtain the EAC in Equation (2).

### 2.3. Setup

**Figure 2:**
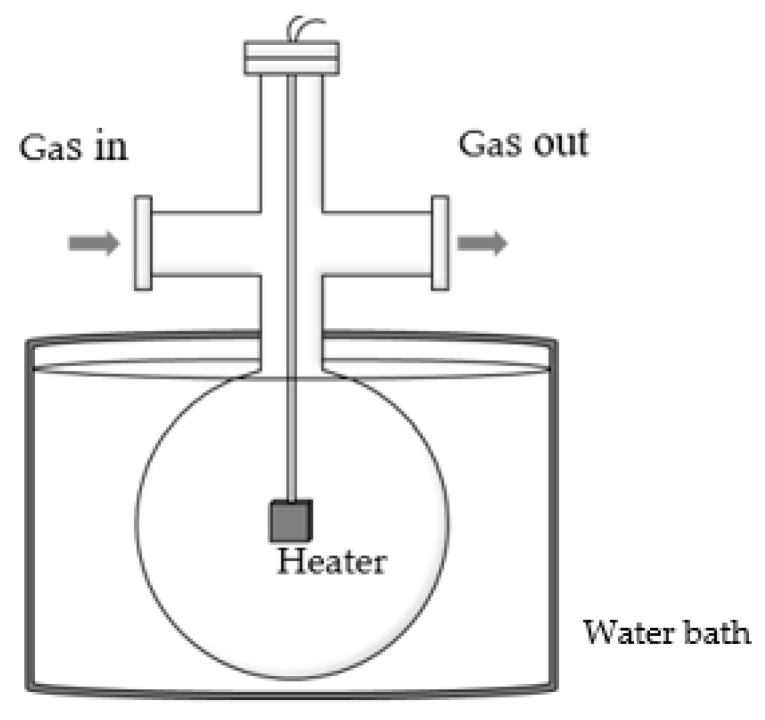
A schematic of the experimental setup.

The experimental setup is explained in detail elsewhere [7,8,9]. A schematic of the experimental set up is shown in Figure 2. A spherical vacuum chamber made by Pyrex, which had a similar shape to a spherical flask, was employed. The inner radius of the chamber $R_{C}$ was 49.5 mm. The chamber was immersed in a water bath to keep the temperature of the chamber $T_{C}$ constant. The measured temperature of the chamber surface $T_{C}$ was about 290 K. The chamber was equipped with NW16 flanges for connections without leakage. The test gas was supplied from commercially available gas cylinders of pure helium and argon. The pressure in the chamber was measured by a temperature-controlled capacitance manometer (Baratron^®^ 627B, MKS Instruments, Andover, MA, USA). The chosen pressure conditions were limited to below 1.4 Pa to be in the near free-molecular flow regime, so that the effect of the general model expression of Equation (3) was minimized.

The sample surfaces of the anodic oxide films were placed on both sides of a tiny flat-shaped heater with the size of 11.8 × 12.0 × 0.38 mm^3^ (Toyo Precision Parts MFG, Nara, Japan). The heater with the sample surfaces was placed at the center of the spherical vacuum chamber. Since the heater was small compared with the spherical vacuum chamber, the temperature in Equation (2) could be approximated by the temperature of the chamber $T_{C}$ due to the large surface area ratio [7,8,9]. It also appeared that the system could be approximated as a concentric spherical shells system for estimating the heat flux in the continuum limit in Equation (3) [7,8,9]. The heat flux in the continuum limit $q_{C}$ was calculated by approximating the sample surfaces as a sphere with radius $R_{H}$ having the same surface area, and the expression becomes as

(4) $$q_{C}=\kappa \left(T_{C}\right)\frac{T^{\omega +1}-1}{\left(\omega +1\right)\left(T-1\right)}\Delta T\frac{R_{C}R_{H}}{R_{C}-R_{H}}\frac{1}{R_{H}{}^{2}},$$

where κ(T), ω, T are the thermal conductivity of the gas at temperature T, the thermal conductivity index, and the temperature ratio of two surfaces $T_{H}/T_{C}$, respectively. The thermal conductivity was assumed to be proportional to $T^{\omega }$ following the model with the inverse power law potential for a monatomic gas. On the other hand, the modified expression, which slightly modifies the heat flux in the continuum in Equation (3), was obtained for better fitting to the results of the S-model solutions as [8]

(5) $$\frac{1}{q}=\frac{1}{q_{FM}}+\frac{1}{\zeta \;q_{C}},\;\zeta =\frac{1}{1-\frac{c_{1}}{\delta_{0}+c_{2}}},$$

where $\delta_{0}=\frac{R_{C}-R_{H}}{l}$, $l=\frac{\mu \left(T_{C}\right)\bar{v}}{p}$, which is the equivalent mean free path, μ is the viscosity, $c_{1}=1.04\alpha \frac{T_{H}}{T_{C}}\frac{R_{H}}{R_{C}}$, $c_{2}=1.97\alpha \frac{T_{H}}{T_{C}}\frac{R_{H}}{R_{C}}$, respectively. We also tried to employ this expression. It was suggested that Equation (5) gave a smaller EAC than Equation (3). The thermal conductivity and viscosity were obtained from [9,12].

An analog electrical bridge circuit was employed to maintain the temperature of the heater by keeping the resistance of the heater element printed by platinum paste constant. The heat transfer rate from the sample surfaces was measured by an electrical consumption to keep the heater temperature constant. The energy consumption consisted of the heat conduction through gas, which we wanted to measure, the radiation and the heat loss through the electrical leads of the heater. Since only the first term depended on pressure, this term could be extracted by evaluating the latter two terms by extrapolating the heat flux to the vacuum limit using values below 0.1 Pa. The convection was negligible due to the low pressure condition. The heat flux q was calculated from the heat transfer rate and the surface area of the samples. The temperature of the sample surfaces was estimated from the electrical resistance of the heater. The calibration curve between temperature and the electrical resistance of the heater with sample surfaces was measured beforehand. The sample surface temperature $T_{H}$ was about 360 K.

The uncertainty of the measurement was quite difficult to evaluate; however, the error of the measurement was known to be less than 5% [7,8,9]. Therefore, it was possible to compare the results at least qualitatively between the conditions.

## 3. Results and Discussions

### 3.1. Heat Flux

**Figure 3:**
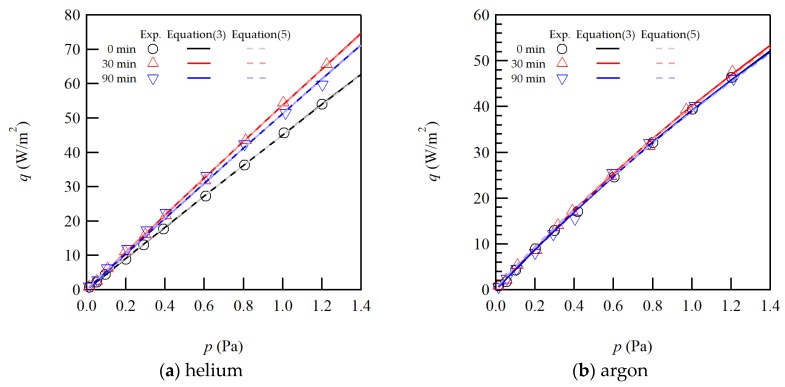
Typical results of the measured heat fluxes and the fitted curves as a function of pressure for 0 min, 30 min and 90 min sample surfaces for (**a**) helium and (**b**) argon.

The heat flux as a function of pressure was measured four times for each condition to check the repeatability. Typical examples of the measured heat fluxes and the fitted curves by Equation (3) and Equation (5) for the 0 min, 30 min and 90 min sample surfaces are plotted in Figure 3 for helium and argon. From the figure, the experimentally measured data are well explained by the fitting curves.

For the three surface samples, the sizes of the heat flux are clearly different for helium; while they are almost similar for argon. It is well known that helium is quite sensitive to the surface conditions due to its light molecular mass and small size. From the figure for helium, the gradient of the heat flux increases by the anodization. However, it slightly decreases when the anodizing time increases up to 90 min. If we close up the results for argon, also the same trend for the three surface samples is observed.

### 3.2. Energy Accommodation Coefficient

**Figure 4:**
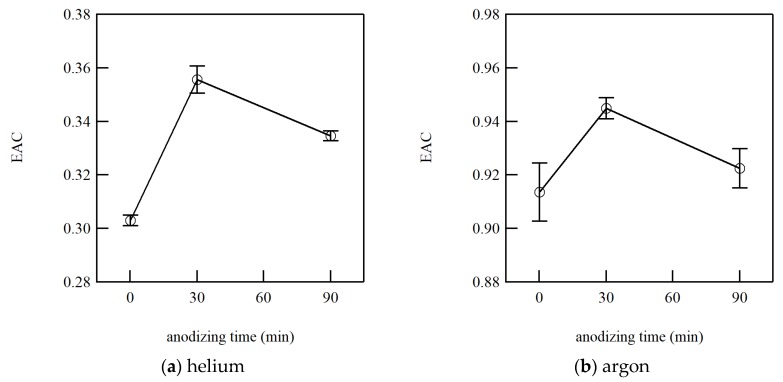
The measured EAC by Equation (5) as a function of the anodizing times of 0 min, 30 min and 90 min for (**a**) helium and (**b**) argon. Error bars show the standard error of 4 measurements for each condition.

The EAC was calculated from Equation (2) by fitting the measured heat flux with the curve of Equations (3) and (5). The EAC was obtained for each condition, i.e., four for each gas sample surface pair. The standard error of the EAC was calculated to evaluate the repeatability of the measurements. The size of the standard error could be an idea for the uncertainty of the measurement. The averaged EACs with the standard error are tabulated in Table 1. From Table 1, the EAC from Equation (3) is slightly larger than that from Equation (5), as mentioned above. Meanwhile, they are qualitatively in good agreement. Thus, only the EAC from Equation (5) is plotted with the error bars representing the standard error in Figure 4 for helium and argon.

From Figure 4, the EAC appears to increase at the anodizing time of 30 min, while it decreases for 90 min. Compared with Figure 3, it is easy to understand the same trend with the gradient of the heat flux curve, since the heat flux is proportional to the EAC in the free molecular regime as in Equation (2), though it is much easier to see the difference in Figure 4. The error bars for argon seem to be much larger than those for helium. However, the absolute value of the EAC for argon is almost three times of that for helium and the relative errors are almost in the same range at less than 1.5%. Then, the measurement accuracy appears to be independent of gas species, and the heat flux is measured with reasonably good accuracy.

Comparing the results of 0 min and 30 min sample surfaces, the EAC is clearly shown to increase by anodization of an aluminum surface. There could be two reasons for this increase: the formation of the nano-scale roughness and the oxidization of aluminum surface, i.e., the difference in materials. For 90 min sample surfaces, the surface was oxidized but is much smoother because of the dissolution of the nano-scale hexagonal cylindrical cell structure of the anodic oxide film on aluminum, as mentioned above. Therefore, we consider that the former effect accounted for the difference between the 30 min and 90 min surface samples; while, the latter effect was the difference between the 0 min and 90 min surface samples. Even though it was difficult to observe the cell structure in our SEM images in Figure 1, the nano-scale roughness was formed on the 30 min sample surfaces, and it will increase the EAC for about 0.02. This qualitative trend is coincident with the well-known characteristics of the EAC.

In [4], it is stated that the effect of macroscopic surface roughness plays only a minor role in the EAC by comparing the results on the machined and the polished surfaces for argon, nitrogen and helium. Comparing the results for the machined and polished 304 Stainless steel surfaces, the EAC decreased from 0.46 to 0.42 for helium, gave the same value of 0.87 for nitrogen, and increased from 0.95 to 0.96 for argon. In our study, the variation size in the EAC for the change of roughness was similar; however, the EAC was increased for both helium and argon. The difference between this study [4] and our study could be coming from the approach to roughening a sample surface. In [4], the machining process was employed for changing the roughness. The surface morphology was modified only for accessible areas from outside. Whereas, the anodization process, which is a wet process, was employed in our study, and it can modify the whole surface area where gas molecules approach. Therefore, the EAC increased for both gas species. It is reasonable to consider that the roughness increases the EAC, even though the size of variation is not large.

## 4. Conclusions

The heat flux from an anodic oxide film on aluminum was measured in the free-molecular to near free-molecular flow regime. The sample surfaces were prepared for three conditions: without anodization (0 min), and with the anodizing times of 30 min and 90 min. For our anodization conditions, 90 min was too long, and a part of the film would be dissolved. SEM images were taken for the three sample surfaces: 0 min, 30 min and 90 min. The surface seemed roughened by the anodization, but it was relatively smoothened and became powdery for 90 min sample surfaces. We failed to capture the detailed hexagonal cylindrical cell structure; however, the formation of the film was validated by an electrical resistance.

The heat flux from the sample surface was measured, and the obtained heat flux was fitted by the curve to extract the energy accommodation coefficient. The obtained energy accommodation coefficient was larger for the 30 min sample surface than for the 0 min or 90 min sample surface, indicating that the EAC increased with an increase in the surface roughness by the anodization process. The decrease of the EAC from the 30 min to 90 min sample surfaces indicated the dissolution of the anodic oxide film on aluminum, and coincided with the relatively smooth surface observed in the SEM image. These characteristics were observed for both helium and argon.

## Figures and Tables

**Table 1:** A table of the measured energy accommodation coefficient (EAC) with the standard error on 0 min, 30 min and 90 min sample surfaces for helium and argon.

Used Equation	Gas Species	0 Min	30 Min	90 Min
Equation (3)	Helium	0.3049 ± 0.0020	0.3582 ± 0.0051	0.3369 ± 0.0018
	Argon	0.9347 ± 0.0115	0.9675 ± 0.0039	0.9440 ± 0.0077
Equation (5)	Helium	0.3030 ± 0.0020	0.3556 ± 0.0051	0.3346 ± 0.0018
	Argon	0.9135 ± 0.0108	0.9449 ± 0.0039	0.9225 ± 0.0074
